# Social contact patterns in Japan in the COVID-19 pandemic during and after the Tokyo Olympic Games

**DOI:** 10.7189/jogh.12.05047

**Published:** 2022-12-03

**Authors:** Shinya Tsuzuki, Yusuke Asai, Yoko Ibuka, Tomoki Nakaya, Norio Ohmagari, Niel Hens, Philippe Beutels

**Affiliations:** 1 Disease Control and Prevention Center, National Center for Global Health and Medicine, Tokyo, Japan; 2 Centre for Health Economic Research and Modelling Infectious Diseases, Vaccine & Infectious Disease Institute, Faculty of Medicine and Health Sciences, University of Antwerp, Antwerp, Belgium; 3 Faculty of Economics, Keio University, Tokyo, Japan; 4 Graduate School of Environmental Studies, Tohoku University, Sendai, Japan; 5 Interuniversity Institute for Biostatistics and statistical Bioinformatics, Data Science; Institute, Hasselt University, Hasselt, Belgium

## Abstract

**Background:**

Social contact data in Japan have not been updated since 2011. The main objectives of this study are to report on newly collected social contact data, to study mixing patterns in the context of the COVID-19 pandemic, and to compare the contact patterns during and after mass events like the 2020 Olympic Games, which were held in 2021.

**Methods:**

We compared the number of contacts per day during and after the Olympic Games and on weekdays and weekends; we also compared them with a pre-COVID-19 pandemic social contact study in Japan. Contact matrices consisting of the age-specific average number of contacted persons recorded per day were obtained from the survey data. Reciprocity at the population level was achieved by using a weighted average.

**Results:**

The median number of contacts per day was 3 (interquartile range (IQR) = 1-6). The occurrence of the Olympic Games and the temporal source of data (weekday or weekend) did not change the results substantially. All three matrices derived from this survey showed age-specific assortative mixing patterns like the previous social contact survey.

**Conclusions:**

The frequency of social contact in Japan did not change substantially during the Tokyo Olympic Games. However, the baseline frequency of social mixing declined vs those collected in 2011.

Mixing patterns in the population are key determinants for explaining the spread of infectious diseases and for assessing the possible impact of non-pharmaceutical interventions like school closure, travel restrictions, and city lockdowns on outbreaks of emerging infectious diseases transmitted from human to human through the respiratory or close-contact route, like COVID-19 [1-8].

Since Mossong et al. constructed social contact matrices of European countries from the POLYMOD contact survey [9], they have been utilized in many studies [10-13].

Social mixing patterns differ by country and change over time. For instance, Ibuka et al. [14] developed a social contact matrix based on a questionnaire survey of the Japanese general population conducted in 2011. They reported that the Japanese population had a greater frequency of contacts than Europeans, although the overall age-specificity of the mixing patterns was similar. According to Prem et al., contact matrices for children in African countries showed more frequent contact among children than for those in European countries, although they showed similar age assortativity [15]. Additionally, the timing of the survey might potentially determine its results, even in the same country. A previous study from Belgium did not show fundamental differences in contact patterns between 2006 and 2010/2011 [16]. Two surveys from Hong Kong showed a large difference in the frequency of contact between two seasons (8.1 in the 2015/2016 and 18.0 in the 2009/2010 season) [17,18].

Coronavirus disease 2019 (COVID-19) caused by the SARS-CoV-2 virus has become a global threat to public health [19,20]. In Japan, as in other countries, various non-pharmaceutical interventions have been implemented in the early stage of the COVID-19 pandemic, including school closure, reduced opening hours in restaurants and bars, and the promotion of remote working. [21]. Consequently, the daily social behaviour of the Japanese population changed drastically. The government repeatedly declared a state of emergency and recommended avoiding “Three Cs (closed spaces, crowded places, and close-contact settings)” [22]. Given the circumstances, we hypothesized that the number of contacts in Japan is expected to have decreased compared to the pre-COVID-19 pandemic period. While European countries have already updated their information about social mixing patterns [23], there has been no updated information in Japan since 2011. The first objective of this study is to update the social mixing patterns of Japan in the context of the COVID-19 pandemic.

Furthermore, we should note that Japan organised the Tokyo 2020 Olympic Games, which were held in 2021 [24]. The event can be regarded as one of the largest scale mass gatherings during the pandemic, presenting a greater risk for the spread of COVID-19 [25-27]. Nevertheless, we have very little quantitative evidence about how social mixing patterns vary by large international mass gathering events such as the Olympic Games, though we hypothesise that the Games increased the frequency of contact. Therefore, the second objective of this study is to compare the mixing patterns and frequency of social contact in Japan during and after the Tokyo Olympic Games.

## METHODS

### Study population and data

We conducted an online survey between August 4, 2021, and August 17, 2021. The participants were voluntarily and randomly recruited from registrants (respondents) of INTAGE RESEARCH INC, a Japanese marketing research company. The same number of invitation emails was sent to the registrants in both survey periods, “during” the Olympic Games period (August 4 – August 9) and “after” the Olympic Games period (August 10 – August 17). Each period included one set of weekends (Saturday and Sunday). In addition, participants were recruited according to quota for age, gender (sex-ratio = 1), and population in each prefecture based on Japan’s 2015 census. An additional survey was conducted for obtaining more detailed information about the age of persons they contacted on the day of the first survey (i.e., between August 4 and August 17, 2021) between September 10 and September 13, 2021 We included only participants who responded to both surveys (n = 3337). Among them, 1953 were enrolled during the Tokyo Olympic Games (between July 23, 2021, and August 8, 2021) and 1384 responded to the survey after the Olympic Games were closed.

Following the previous study on social contacts in Japan [14], respondents answered survey questions online, about social contacts for themselves and for household members who were under the age of 20 at the time of the survey. Those asked about their household members were given the option of taking a break to consult with their household members before specifying contacts of household members. We defined respondents and participants separately. Respondents were individuals who answered the survey directly and participants were respondents’ household members who did not answer the questionnaire directly. For example, if a mother responded on behalf of a child, the mother was a respondent while the child was a participant. Participants were instructed to make their best guess when they did not know the exact information about the age of their contacts.

Information about the participants’ basic demographics and each age group’s frequency of contacts was collected. The survey was conducted as a single-day point prevalence survey, like many other contact studies, including the study by Ibuka et al. [14]. As a result, respondents gave the details of their contacts on the day preceding the one on which they completed the questionnaire. The English version of the questionnaire is available in section 1 of the **Online Supplementary Document**.

A contact was defined as 1) a conversation of three or more sentences within two meters distance, 2) a direct conversation with others (indirect ones such as via telephone were excluded), 3) conversations with face coverings or partitioning, 4) a dinner with other people, where all those present at the table are considered contacts, 5) more than one conversation with the same person (counted as one contact), and 6) physical contact with a person (counted as one contact). Thebasic definition of a contact was similar to the previous study but explanations about face covering, partitioning, and how to count group contacts were added.

### Sample size calculation

First, we assumed that the frequency of contact in this study had decreased compared to previous studies by Ibuka et al. [14]. In this case, we have no data about standardized differences between the data obtained by the previous study and the ones obtained by our survey. Therefore, we set the power at 0.9 and the effect size at 0.2, constituting a small difference [28,29]. Consequently, power calculation by a student’s *t* test demonstrated that 527 samples in each group (the previous study and the present study) were required.

Next, we assumed that the frequency of contacts in the weekends was smaller than that on weekdays, as the previous study reported. We set the power at 0.9 and the effect size (Cohen’s d) of weekends was calculated as 4.26 from the previous study [14]. This difference can be regarded as “huge”, and therefore we set the effect size at 0.8 so as not to overlook smaller differences between weekdays and weekends [28,29]. As a result, power calculation by a student’s *t* test demonstrated that 34 samples in each group (weekdays and weekends) were required.

### Data analysis

The descriptive analysis of the participants’ basic demographic characteristics is presented with continuous variables summarised by their median and interquartile range (IQR) and factors of categorical variables by their absolute number and percentage. The normality of continuous variables was examined by the Shapiro-Wilk test, yielding a non-normal distribution for all continuous variables presented in the results.

Factors associated with the number of contacts were examined using random forests [30], which is a class of ensemble methods that generate many classifiers or predictions and aggregate their results, specifically designed for classification or regression trees. We used the feature selection algorithm from the Boruta package [31] in R for constructing a variable importance list. The Boruta algorithm is a wrapper implemented in the R package randomForest [32]. The details of its algorithm are described in Section 2 of the **Online Supplementary Document**.

Further, we examined the relative number of contacts in different age groups, between genders, during or after the Olympic Games, and among other factors selected by the Boruta process by a negative binomial regression model. Two-sided *P* values of <0.05 were considered statistically significant.

### Contact matrices

We established contact matrices from the questionnaire data consisting of the average number of contact persons recorded per day. Reciprocity was obtained by averaging the population-level number of contacts of the corresponding cells [33,34]. Additionally, we made another contact matrix based on the year 2011 data derived from Ibuka et al. [14] For this pre-COVID-19 matrix, we determined a weighted average *d_ij* of the number of contacts in age group *j* made by participants of age group *i*. We made four matrices using our year 2021 data; a weekday (from Monday to Friday) matrix (W), a weekend (Saturday and Sunday) matrix (H), a matrix after the Olympic Games (A), and a matrix during the Olympic Games (O). This gave the elements of the contact matrix *φ_ij = c_ij*, scaled by the time period T over which contacts were measured (in this study, per day then *t* = 11). All analyses were conducted using R, version 4.1.2 [35].

## RESULTS

The basic characteristics of the participants are shown in **Table 1**. As described in the previous section, 1953 participants were enrolled during the Olympic Games, while 1384 were enrolled after the Olympic Games. Among them, 1600 (47.9%) were male and 1713 (51.3%) were from urban areas. 2198 (65.9%) were enrolled on weekdays. During the Olympic Games, only 79 (4.0%) went to the venue and 26 (1.3%) watched the games at sports bars, because many of the games took place without live spectators.

**Table 1:** Basic characteristics of the respondents

	During the Olympic games (n = 1953)	After the Olympic games (n = 1384)	Total (n = 3337)
**Age in years (IQR)**	37 (12-50)	37.5 (14-51)	37 (13-50)
**Male**	970 (49.7%)	630 (45.5%)	1600 (47.9%)
Residents in urban area*	980 (50.2%)	733 (53.0%)	1713 (51.3%)
**Working status**			
Full time	1062 (54.4%)	729 (52.7%)	1791 (53.7%)
Part time	349 (17.9%)	220 (15.9%)	569 (17.1%)
Job seeking	50 (2.6%)	41 (3.0%)	91 (2.7%)
Others (students etc.)	492 (25.2%)	394 (28.5%)	886 (26.6%)
**Education**			
Secondary	45 (2.3%)	25 (1.8%)	70 (2.1%)
High school	623 (31.9%)	414 (29.9%)	1037 (31.1%)
University	1277 (65.4%)	938 (67.8%)	2215 (66.4%)
Others	8 (0.4%)	7 (0.5%)	15 (0.4%)
**Number of household members (IQR)**	3 (2-4)	3 (2-3)	3 (2-3)
**Participated in weekdays**	1518 (77.7%)	680 (49.1%)	2198 (65.9%)
**Remote working on the day of survey**	173 (8.9%)	57 (4.1%)	230 (6.9%)
**Watching the Olympic Games**	-	NA	NA
On site	79 (4.0%)	-	-
At sports bar	26 (1.3%)	-	-
**Commute to workplaces**	1246 (63.8%)	840 (60.7%)	2086 (62.5%)
**Frequency of dining out per month**			
Before pandemic (IQR)	1 (0-2)	1 (0-3)	1 (0-2)
During pandemic (IQR)	0 (0-0)	0 (0-0)	0 (0-0)

NA – not available

*Ordinance-designated cites, prefectural capital and other cities of similar size.

The random forests approach using the Boruta method showed that age was the most important factor influencing the total number of contacts made. Additionally, contacts at work and permittance of remote work were also important. Notably, the timing of the survey (weekday or weekend, during or after the Olympic games) was not found to be an influential factor. Visiting a sports bar or being onsite during the Olympic Games were also not influential. The results of the random forest approach are described in Figure S1 (section 2) in **Online Supplementary Document**.

**Figure 1:**
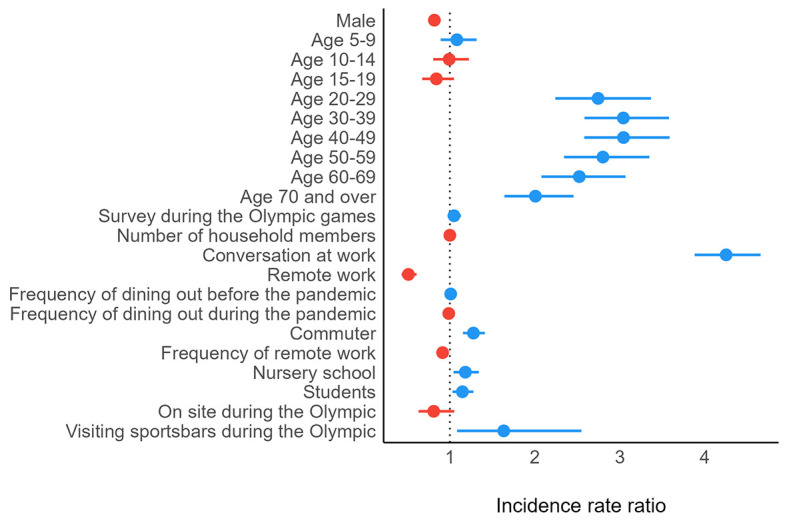
Results of a negative binomial regression model for the total number of contacts. Blue circles represent a positive impact on the total number of contacts. Red circles represent a negative impact on the total number of contacts. The lines on either side of the circles represent 95% confidence intervals.

The results of a negative binomial regression model for the total number of contacts are presented in **Figure 1**. People younger than 20 years of age reported a smaller number of contacts compared with adults. Participants who visited sports bars during the Olympic Games reported a larger number of contacts, but those who visited the site of the Olympic Games reported a lower number of contacts.

The median and mean number of contacts per day was 3 (IQR = 1-6) and 8.92 (standard deviation (SD) = 25.45), respectively. Whether the Olympic Games were held or not, and the timing of survey (weekday or weekend) did not change these results substantially.

**Figure 2:**
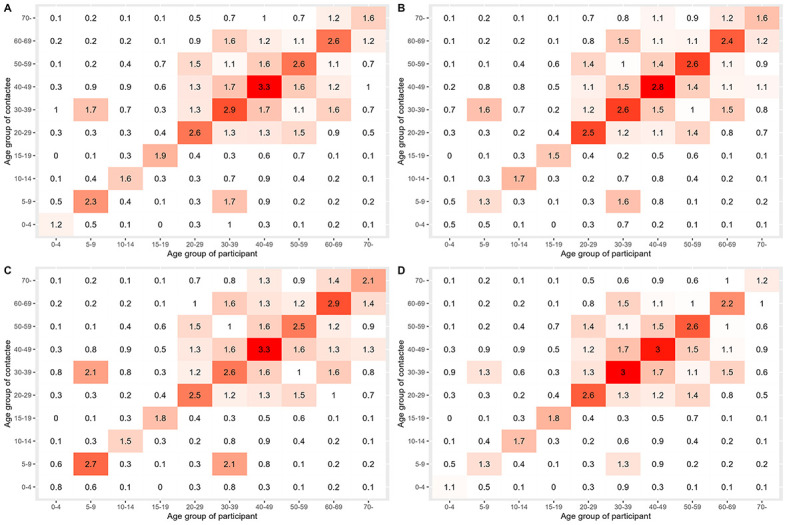
Contact matrices. **Panel A:** Contact matrix based on the weekday survey data. **Panel B:** Contact matrix based on the weekend survey data. **Panel C:** Contact matrix based on the survey data “after the Olympics”. **Panel D:** Contact matrix based on the survey data “during the Olympics”. The red-coloured cells indicate a higher number of contacts compared to white cells. The darker the shade of red, the higher the number of contacts.

**Figure 2** (Panel A-D) and Figure S2 in **Online Supplementary Document** **2** are social contact matrices based on the weekday survey data, the weekend survey data, the survey data “after the Olympic Games” period, the survey data “during the Olympic Games” period, and a re-constructed matrix derived from Ibuka et al. [14]. All four matrices derived from this survey showed an age-specific assortative mixing pattern like the re-constructed matrix derived from Ibuka et al [14]. While the latter matrix showed more frequent contact among children than among adults, our survey results showed the opposite. Furthermore, it was difficult to find obvious differences among the four matrices derived from this survey.

## DISCUSSION

To the best of our knowledge, this is the first study to explain the change of social mixing patterns during and after the Olympic Games in the context of the COVID-19 pandemic.

One of our hypotheses that the COVID-19 pandemic decreased the frequency of contacts in Japan seems reasonable because the average number of contacts per day shown by this study was substantially smaller than that reported by the previous study [14].

Meanwhile, large-scale mass gathering events such as the Olympic Games are expected to increase the chance of social contact among the general population [36,37]. However, our findings demonstrated that their frequency of contact did not change significantly during or after the Olympic Games period. This might be explained by the fact that the Tokyo Olympic Games were held under strict conditions with hardly any live spectators. For instance, traffic regulation was strengthened around Tokyo and the tolls for the Metropolitan Expressway were raised during the Olympic Games. These interventions could have possibly contributed to reducing human flow.

The number of contacts among children was lower than in the previous study (1.6 vs 12.9 in the 10-14 age group) because of the summer vacation period in August. However, even if we exclude children from the survey data, the frequency of social contacts among Japanese adults was substantially lower compared to the pre-COVID-19 period (the median number of contacts per day was 3 vs 12, respectively) [14]. This large difference might be attributed to the current COVID-19 pandemic even when taking the weekend effect into consideration because the difference in contacts between weekdays and weekends in Japan was not that large (14 and 8, respectively) [14]. Since April 10, 2021, the Japanese government declared the third state of emergency for four prefectures including, the Tokyo metropolitan area [38]. After that, the declaration was made for some other prefectures, only to be lifted on June 20 for all, except for the Okinawa Prefecture. However, the state of emergency was declared for Tokyo again on July 12, and the Tokyo metropolitan area had been under a state of emergency until the end of September. The Tokyo Olympic Games were conducted from July 23 to August 8, 2021, and therefore all of the games were done during the declaration [38]. Considering these conditions, the state of emergency declaration may have had a substantial impact on social mixing behaviour of the Japanese general population, although the declaration did not imply legal enforcement.

Our results also showed that weekends did not have a clear influence on mixing behaviour in the COVID-19 pandemic period. This finding can also be attributed to the declaration because it recommended avoiding unnecessary outings and trips. The random forest analysis supports this hypothesis, showing that the most important factor for the increase in the number of contacts other than age was contacts at work. The variation in contacts was determined by work-related behaviour, and not substantially by contacts in the private sphere.

Our study has several limitations. First, it included only the participants who answered two rounds of online surveys. This may impair the reliability of the results because the second online survey was conducted after a month has passed since the Olympic Games had finished, possibly complicating comparisons with similar studies [39,40] due to differences caused by people not participating in the second round. Nevertheless, the survey design can be regarded as appropriate for the study’s main objective since the previous study in Japan was also reported based on the point prevalence survey [14]. Second, our survey was fully internet-based, implying participants had to have basic knowledge of the internet, which could lead to selection bias. However, as most previous studies reported, young adults and children are the main sources of frequent contacts, and thus the selection bias might be less influential. Additionally, the Hoang et al.’s systematic review of contact studies pointed out that no clear relationship in the number of contacts had been found when comparing online diaries with paper diaries [41]. Third, like other previous studies, we could not obtain information directly from children. In our survey, we requested respondents who had children younger than 20 years old living in their household to indicate the number of contacts made by their children, which could lead to biases in reporting, especially in younger children who are primary school students or utilize nursery schools/kindergartens. Fourth, we did not consider the effect of seasonality, vacation, other non-pharmaceutical countermeasures, and other factors. Since August is the summer vacation season in Japan, it is likely that their mixing behaviour is different from other seasons. Furthermore, many Japanese adults take “Obon” vacation in the latter half of August, which exactly corresponds to the period just after the Olympic Games. Further study would be desirable to assess the effect of these social factors on the number of social contacts.

## CONCLUSIONS

The frequency of social contacts in Japan did not change substantially during the Tokyo Olympic Games. However, the baseline frequency of social mixing decreased compared with that reported previously, and this might be attributed to the COVID-19 pandemic and the state of emergency declaration.

## Additional material:


Online Supplementary Document
