# Supplementary material for: Social contact patterns in Japan in the COVID-19 pandemic during and after the Tokyo Olympic Games
Source: J Glob Health. 2022 Dec 3;12:05047. doi: 10.7189/jogh.12.05047 (PMC9718444; doi:10.7189/jogh.12.05047)
Supplement: Online Supplementary Document [file jogh-12-05047-s001.pdf]

ONLINE SUPPLEMENTARY DOCUMENT
Title: Social contact patterns in Japan in the COVID-19 pandemic during and after the Tokyo Olympic Games
Authors: Shinya Tsuzuki, Yusuke Asai, Yoko Ibuka, Tomoki Nakaya, Norio Ohmagari, Niel Hens, Philippe Beutels

Online Supplementary Document 1:

English translation of the questionnaire

As a preliminary step, you will be asked to view the page on explanation and consent for the study, and only monitors who click on the "I agree" button will be able to reach the original questionnaire.

First, let me ask you about yourself.

1. Please tell us your age.

2. Please tell us your gender.

3-a. Please indicate your current prefecture of residence.

3-b. Please indicate your current municipality of residence.

4. Do you currently have a job that pays an income?

(a) Yes (full-time)

(b) Yes (part-time)

(c) No (looking for a job)

(d) No (currently in school, retired, full-time housewife/husband, etc.)

5. If you answered "Yes (full-time)" or "Yes (part-time)" to the previous question

Please select one main item regarding the contents of your job (occupation).

(a) Management (company director, manager, etc.)

(b) Professional/technical (doctor, technician, teacher, etc.)

(c) Administrative (clerks, receptionists, etc.)

(d) Sales (salespersons, sales clerks, etc.)

(e) Service (beauticians, cooks, nursing staff, etc.)
(f) Security (police officers, security guards, etc.)
(k) Agriculture, forestry and fishery (agriculture, forestry, fishery workers, etc.)
(k) Production, construction, transportation (assembly workers, drivers, cleaners, etc.)
(k) Unclassifiable/other than the above

6. Please indicate the school you last graduated from.
(a) Junior high school
(b) High school, special training school
(c) Junior college, vocational school, university/graduate school (science, engineering, medicine, dentistry, agriculture, biology)
(d) Junior colleges, vocational schools, universities and graduate schools (faculties other than the above)
(e) Other

7-a. Please indicate the number of family members living with you.
7-b. Please indicate the age of your family members living with you.

8-a. In your profession, do you routinely converse with an unspecified number of people?
8-b. On average, how many people (customers, patients, students, etc.) do you talk to in a day (from 0:00 to 24:00) in the course of your work?

9. Approximately which of the following age groups do the people you converse with on the job belong to?
a. 0-4
b. 5-9
c. 10-14
d. 15-19
e. 20-29
f. 30-39
g. 40-49
h. 50-59
i. 60-69
j. 70-79

k. 80-89

l. 90 and over

10. We would like to ask you about people with whom you had conversations outside of work yesterday.

First, which of the following dates is 'yesterday' for you?

(You can choose one of between 3rd to 23rd August)

11. How many people did you have face-to-face conversations with between midnight and midnight yesterday?

12. Approximately which of the following age groups do the people you converse with outside the job belong to? Please answer for each person.

a. 0-4

b. 5-9

c. 10-14

d. 15-19

e. 20-29

f. 30-39

g. 40-49

h. 50-59

i. 60-69

j. 70-79

k. 80-89

l. 90 and over

13. Was yesterday a telework or online meeting, study or lecture day?

14. Yesterday, did you watch the Tokyo Olympic Games at your venue?

15. Yesterday, did you watch the Tokyo Olympic Games in a sports bar or other place where an unspecified number of people gathered (other than a venue where you could watch the games)?

16. Before the outbreak of COVID-19, how many times a month did you have the opportunity to meet or drink with others?

17. After the outbreak of COVID-19, how many times a month did you have the opportunity to meet or drink with others?

18. Do you currently commute to work or school?

19. How often do you currently telework, hold online meetings, study/lectures, etc.? Please indicate how many days per week.

20. Now we would like to ask you about your minor child (or youngest child if you have more than one) who live with you.
Please indicate the sex and age of your child

21. Does your child use group childcare such as nursery school or kindergarten?

22. Please indicate the approximate number of children in the class of your child.

23. Does your child attend school?

24. Please indicate the approximate number of children in the class of your child.

25. How many people did your child have face-to-face conversations with between midnight and midnight yesterday?

26. Approximately which of the following age groups do the people your child converse with belong to? Please answer for each person.
a. 0-4
b. 5-9
c. 10-14
d. 15-19
e. 20-29
f. 30-39

g. 40-49

h. 50-59

i. 60-69

j. 70-79

k. 80-89

l. 90 and over

Online Supplementary Document 2: methodological details

The Boruta algorithm consists of following steps [1];

1. Extend the information system by adding copies of all variables (the information system is always extended by at least 5 shadow attributes, even if the number of attributes in the original set is lower than 5).
2. Shuffle the added attributes to remove their correlations with the response.
3. Run a random forest prediction on the extended information system and gather the Z scores computed.
4. Find the maximum Z score among shadow attributes (MZSA), and then assign a hit to every attribute that scored better than MZSA.
5. For each attribute with undetermined importance perform a two-sided test of equality with the MZSA.
6. Deem the attributes which have importance significantly lower than MZSA as ‘unimportant’ and permanently remove them from the information system.
7. Deem the attributes which have importance significantly higher than MZSA as ‘important’.
8. Remove all shadow attributes.
9. Repeat the procedure until the importance is assigned for all the attributes, or the algorithm has reached the previously set limit of the random forest runs.

Figure S1. Feature importance of variables included in the random forest model

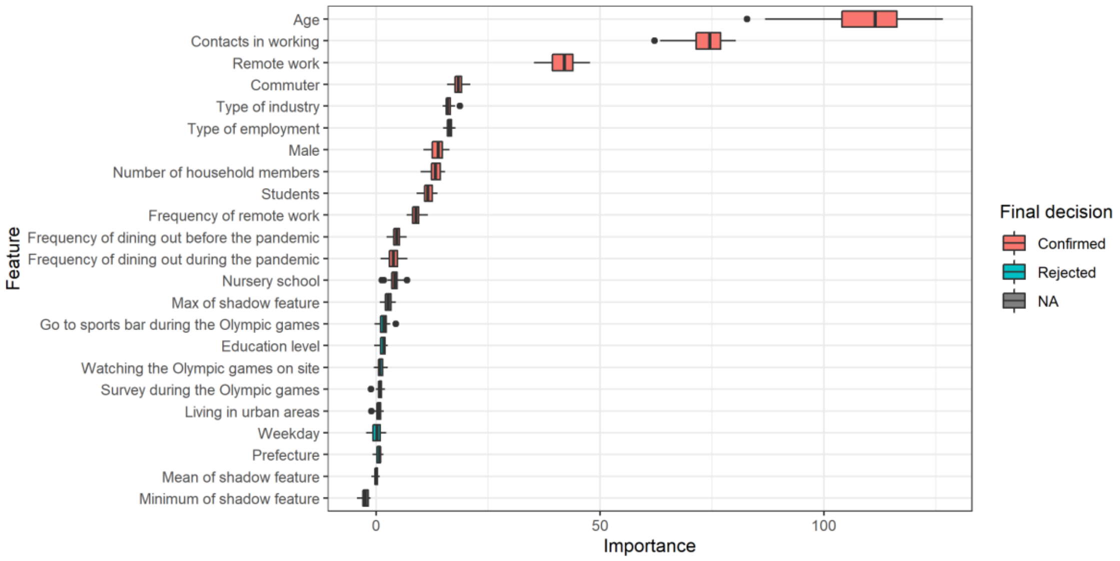

Feature importance was evaluated by mean squared error.

Figure S2. Contact matrix derived from the previous study about social mixing pattern in Japan

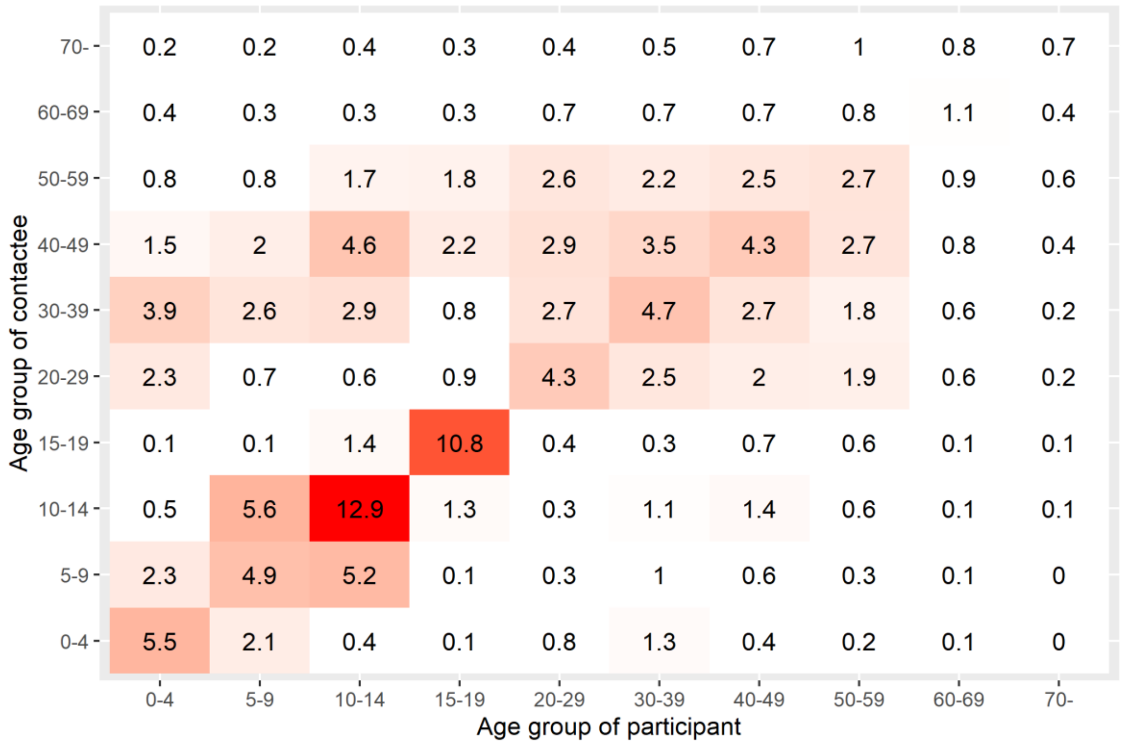

Average number of contacts per day between age groups. Red colour indicates higher contact numbers compared to white cells, with darker colour signifying higher number of contacts.

References

1. Kursa MB, Rudnicki WR. Feature Selection with the Boruta Package. Journal of Statistical Software. 2010;36:1–13.
